# Data-informed debriefing for cardiopulmonary arrest: A randomized controlled trial

**DOI:** 10.1016/j.resplu.2023.100401

**Published:** 2023-05-25

**Authors:** Adam Cheng, Jennifer Davidson, Brandi Wan, Alexandra St-Onge-St-Hilaire, Yiqun Lin

**Affiliations:** a Departments of Pediatrics and Emergency Medicine, Cumming School of Medicine, University of Calgary, KidSIM-ASPIRE Research Program, Alberta Children’s Hospital, 28 Oki Drive NW, Calgary, AB T3B 6A8, Canada; b KidSIM Simulation Program, Alberta Children’s Hospital, University of Calgary, Canada

**Keywords:** Cardiopulmonary arrest, CPR, Debriefing, Data, Simulation

## Abstract

**Aim:**

To determine if data-informed debriefing, compared to a traditional debriefing, improves the process of care provided by healthcare teams during a simulated pediatric cardiac arrest.

**Methods:**

We conducted a prospective, randomized trial. Participants were randomized to a traditional debriefing or a data-informed debriefing supported by a debriefing tool. Participant teams managed a 10-minute cardiac arrest simulation case, followed by a debriefing (i.e. traditional or data-informed), and then a second cardiac arrest case. The primary outcome was the percentage of overall excellent CPR. The secondary outcomes were compliance with AHA guidelines for depth and rate, chest compression (CC) fraction, peri-shock pause duration, and time to critical interventions.

**Results:**

A total of 21 teams (84 participants) were enrolled, with data from 20 teams (80 participants) analyzed. The data-informed debriefing group was significantly better in percentage of overall excellent CPR (control vs intervention: 53.8% vs 78.7%; MD 24.9%, 95%CI: 5.4 to 44.4%, *p* = 0.02), guideline-compliant depth (control vs. intervention: 60.4% vs 85.8%, MD 25.4%, 95%CI: 5.5 to 45.3%, *p* = 0.02), CC fraction (control vs intervention: 88.6% vs 92.6, MD 4.0%, 95%CI: 0.5 to 7.4%, *p* = 0.03), and peri-shock pause duration (control vs intervention: 5.8 s vs 3.7 s, MD −2.1 s, 95%CI: −3.5 to −0.8 s, *p* = 0.004) compared to the control group. There was no significant difference in time to critical interventions between groups.

**Conclusion:**

When compared with traditional debriefing, data-informed debriefing improves CPR quality and reduces pauses in CPR during simulated cardiac arrest, with no improvement in time to critical interventions.

## Introduction

Cardiopulmonary resuscitation (CPR) is provided for thousands of children suffering from cardiopulmonary arrests (CPA) each year in North America.^1^ The provision of guideline-compliant basic life support (BLS) and advanced life support (ALS) improves patient outcomes following cardiac arrest.[2], [3] Unfortunately, healthcare providers struggle to consistently perform guideline-compliant chest compressions[4], [5] and advanced life support[6], [7], [8] during in-hospital cardiac arrest. Delays in epinephrine administration[9], [10] and defibrillation[6], [11], [12] represent common deviations from American Heart Association (AHA) resuscitation guidelines associated with poor patient outcomes from cardiac arrest. Effective strategies to optimize BLS and ALS care during cardiac arrest are necessary.

Debriefing is a group learning conversation “in which aspects of performance are explored and analyzed with the aim of gaining insights that will impact the quality of future clinical practice”.^13^ Clinical debriefing conducted after cardiac arrest events improve provider performance,[14], [15] while debriefings informed by clinical data (e.g. CPR quality metrics, time to defibrillation) collected during the cardiac arrest event have been associated with improved survival outcomes from pediatric cardiac arrest.[16], [17] Unfortunately, post-event debriefings are infrequently conducted in most institutions, and when conducted, objective data is rarely used to support these conversations.[18], [19] As a result, resuscitations teams are forced to rely upon provider recall of resuscitation events, which is often flawed and inaccurate,[20], [21] to frame their discussions during debriefings. These debriefing conversations may fail to focus on critical errors, which can directly influence the quality of care provided in the future. The advent of CPR feedback defibrillators that collect objective performance data offers a unique opportunity to address this issue.^17^

Debriefing tools have been used to support facilitation of debriefing conversations by providing structure, sample questions or phrases, and suggested topics for discussion.[22], [23], [24], [25], [26], [27] While their use has gained traction in both educational[22], [23] and clinical settings,[24], [25], [26] the true benefit of debriefing tools supplemented by objective performance data from cardiac arrest events is uncertain. An understanding of the impact of data-informed debriefing with a debriefing tool will assist programs in implementing clinical debriefings that directly impact performance during cardiac arrest. In this study, we aim to assess if data-informed debriefing supported by a debriefing tool, compared to a traditional debriefing (with no objective data or tool), improves the process of resuscitative care and quality of CPR provided by pediatric healthcare teams during a simulated pediatric cardiac arrest.

## Methods

We conducted a prospective, randomized controlled trial. Research ethics board approval was secured and informed consent was obtained from all participants. Our project utilized established simulation-based research methods to address our research objectives.^28^

### Study participants

Healthcare providers from the emergency department and intensive care unit of the Alberta Children’s Hospital were recruited to participate in the study. Participants were consented and recruited in teams of four for the roles of team leader, CPR provider (two participants), and charting nurse. Inclusion criteria for the team leader were: (1) Attending physician or fellow in pediatric / adult emergency medicine, pediatric intensive care, or pediatric anesthesia; and (2) Adult or Pediatric Advanced Life Support certification. Inclusion criteria for the other roles were the same as above, but also included resident doctors, nurses, and respiratory therapists.

### Study procedures

Participants were randomized in teams of four using an online randomizer tool into either the control arm or intervention arm. Randomization occurred at the level of the team. Study packages were prepared with opaque envelopes and administered by a research coordinator to achieve allocation concealment.

Teams were randomized to one of 2 groups: (a) Intervention: data-informed debriefing with use of a debriefing tool; or (b) Control: traditional debriefing with no objective data and no debriefing tool. For each group, two research actors played the roles of airway provider and medication nurse to create a resuscitation team of six healthcare providers (i.e. 2 actors and 4 participants). All actors were trained to portray their role in a standardized fashion by following team leader instructions and contributing input when asked. Actors did not provide any unsolicited advice related to clinical care of the simulated patient and did not provide chest compressions. All teams received a standardized pre-briefing orienting them to the research study, clinical environment, manikin features, and the Zoll R-Series^TM^ Defibrillator with CPR feedback device, which was available to both groups. Following the pre-briefing, all teams participated in two sequential simulation scenarios with a debriefing (control vs. intervention) after the first scenario.

#### Simulation scenarios

Both cardiac arrest scenarios were 10 minutes in duration and tightly standardized by using a scenario template with scripted patient progression. The first simulated cardiac arrest scenario depicted a patient progressing from shock (two minutes) → ventricular fibrillation (four minutes) → pulseless electrical activity (four minutes), while the second scenario depicted a patient progressing from shock (two minutes) → pulseless electrical activity (four minutes) → ventricular fibrillation (four minutes). The case history for the two scenarios was slightly different, but case difficulty and clinical management components were the same between cases. The Zoll R-Series^TM^ Defibrillator with CPR feedback technology collected CPR quality data and time to defibrillation, and the charting nurse recorded time to epinephrine and other clinical tasks. All scenarios were video-recorded from a birds-eye view from the foot of the bed.

#### Data-informed debriefing

Two debriefers (AC and YL) conducted all the debriefings in this study. Both are trained simulation educators and pediatric emergency medicine physicians with over 25 years of collective experience debriefing in educational and clinical contexts. A data-informed debriefing tool was developed, with several phases modeled after the PEARLS blended method of debriefing (i.e. reactions, analysis, summary).[23], [27] The goal of the reactions phase was to collect initial thoughts from the participants and to preview the debriefing by highlighting the role of objective data. In the analysis phase, debriefers systematically reviewed key tasks and associated targets provided on the debriefing tool, along with standardized teaching points for each task that were identified from our prior series of studies exploring team performance during cardiac arrest[29], [30], [31], [32] (Table 1). In contrast to debriefings primarily focused on exploring learner frames, debriefings conducted in the intervention group were heavily focused on objective data and discussing how to achieve performance targets for each task. In the data-informed debriefing group, CPR quality data (mean CPR depth and rate, % compliance with CPR depth and rate, % overall excellent CPR, chest compression fraction [CCF], peri-shock pause duration, other pauses in compressions, and time to defibrillation) was downloaded from the defibrillator and shared via display on a large screen in the debriefing. Time to epinephrine administration and time to definitive airway insertion was collected from the charting nurse records and discussed during the debriefing. Relevant performance data was shared with the group, participants were invited to share their perspective, and then performance targets served as a trigger point for identifying opportunities for improvement. Teaching points were provided via directive feedback when participants did not close performance gaps through discussion. Both facilitators were versed in (or trained in) the Promoting Excellence and Reflective Learning in Simulation (PEARLS) debriefing methodology and were provided opportunity to practice two debriefings with the new debriefing tool prior to study implementation.

**Table 1:** Cardiac Arrest Debriefing Tool.

**Phase**	**Task / Targets**	**Teaching Point**
**Reactions**	Preview debriefing and collect reactions	• Highlight importance of objective data
**Analysis**	**KEY TASKS & TARGETS**	
	*Time to CPR*	
	• Target < 1 min	• Assign roles ahead of time
		• Anticipate need for CPR
		• Initiate CPR before board placement
		• Place board and apply pads at same time
		• Verbalize pulseless rhythm
	*Time to Defibrillation*	
	• Target < 1 min	• Verbalize shockable rhythm
		• Assign / pre-assign task to capable provider
		• Shock immediately, do not delay for epi
	**CPR QUALITY**	
	*Depth*	
	• Target – 5–6cm	• Place backboard early
		• Press hard / deep
		• Ensure full recoil
		• Verbal coaching by other CPR provider / coach
		• Ensure defib screen is visible to provider
		• Coach immediately after each switch
	*Rate*	
	• Target – 100–120/ min	• Verbal coaching by other CPR provider / coach
		• Ensure defib screen is visible to provider
		• Coach immediately after each switch
	*Recoil*	
	• Target – full recoil	• Ensure full recoil
		• Verbal coaching by other CPR provider / coach
		• Ensure defib screen is visible to provider
		• Coach immediately after each switch
	**PAUSES**	
	*Pause Duration*	
	• Target <5 s/ pause	• Coordinate tasks during pauses
		• Team leader shares mental model 15s before pause
		• Prepare next CPR provider
		• Immediate resumption of CPR (verbalize)
	*Peri Shock Pause*	
	• Target < 10 s	• Team leader shares mental model 15s before defib
		• Continuous CPR during charging
		• Prepare next CPR provider
		• Brief verbal guidance to shock
		• Immediate resumption of CPR (verbalize)
		
	*Pauses During Intubation*	
	• Target < 10 s	• Team leader shares mental model 15s before intubation
		• Continuous CPR during intubation attempt
		• Pause (max 10 s) if unable to visualize cords
	*CCF*	
	• Target >80%	• Group tasks during pauses
		• Early intubation to allow continuous CPR
	**TIME TO MEDICATION / AIRWAY**	
	*Time to Epinephrine*	
	• Target < 1 min	• Prepare epinephrine early
		• Insert IO quickly
		• Give immediately once IO inserted
	*Time to Definitive airway*	
	• Target – secure definitive airway early (after defib and epi administration)	• Assign roles
		• Prepare equipment early
		• Continue compressions during procedure
		• Keep pauses in CPR under 10 s
**Transition**	Anything else to discuss?	
**Summary**	Key take home messages – one from each learner	

In the control group, debriefings were conducted by the same two debriefers (AC and YL), who used a PEARLS blended-method of debriefing to facilitate and structure discussion. The debriefers discussed all the same tasks and performance metrics with participants, but objective data was not provided or reviewed. Performance gaps were closed through group discussion with participants in a learner-centered manner^33^; specific feedback and teaching points were provided when requested by participants. All debriefings were capped at 20 minutes in duration. The second scenario was done immediately after the debriefing. Following the second scenario, teams in both groups received a very short educational debriefing which was not part of the intervention.

### Outcome measures

CPR quality parameters including chest compression (CC) depth (cm), CC rate (cc/min), CC fraction (the percentage of time during cardiac arrest with CC) and peri-shock pause duration were collected from the Zoll R-Series^T^^M^ Defibrillator, which has been used in prior clinical and simulation-based studies to report CPR quality.[5], [30], [34], [35], [36] In accordance with previous publications on CPR quality, average CC depth and CC rate were calculated for each one-minute epoch (interval) of resuscitation.[30], [31] Compliance with 2020 AHA guidelines were defined as: depth 50 to 60 mm and rate 100 to 120/min.[37], [38], [39] Three additional outcome measures (i.e. time to initiation of CC, time to epinephrine, time to definitive airway insertion) were captured from video review of the simulation scenarios. Videos were reviewed by a trained and calibrated rater with a background in emergency medicine who was blinded to the group allocation.

*Primary outcome measure*: percentage of overall excellent CPR, defined as meeting AHA guidelines for both CC depth and rate.[1], [38], [39]*Secondary outcome measures*: (i) the percentage of CC meeting AHA guidelines for depth (5–6 cm); (ii) the percentage of CC meeting AHA guidelines for rate (100–120 bpm); (iii) CC fraction; (iv) peri-shock pause duration (seconds); (v) time to first defibrillation (seconds); (vi) time to initiation of CC (seconds); and (vii) time to first epinephrine dose (seconds).

### Sample size

Sample size estimation was based on the primary outcome measure. Prior research conducted by our team shows that the mean percentage of guideline-compliant CPR is approximately 45% with a standard deviation of 15% amongst trained healthcare providers when real-time feedback is provided.^31^ To detect a 20% increase of guideline-compliant CPR with a significance level of 0.05 and power of 0.8, each arm required 9 teams (36 providers), making our total sample size 18 teams (72 providers). To account for 10% missing data due to technique issues, we recruited a total of 21 teams (84 participants).

### Statistical analysis

All analyses were conducted with R software (version 4.2.2. https://www.r-project.org) with a significance level of 0.05. We summarized the demographic characteristics of the 2 groups with descriptive statistics (count and percentage for categorical variables and median and interquartile range [IQR] for numeric variables). The differences between the traditional debriefing and data-informed debriefing groups were compared with 2-sample t-tests for all outcome measures for both pre-debriefing performance and post-debriefing performance. We conducted linear regression models to examine the difference of post-debriefing performance between the groups adjusting for pre-debriefing performance (baseline performance).

## Results

### Demographics

**Fig. 1:**
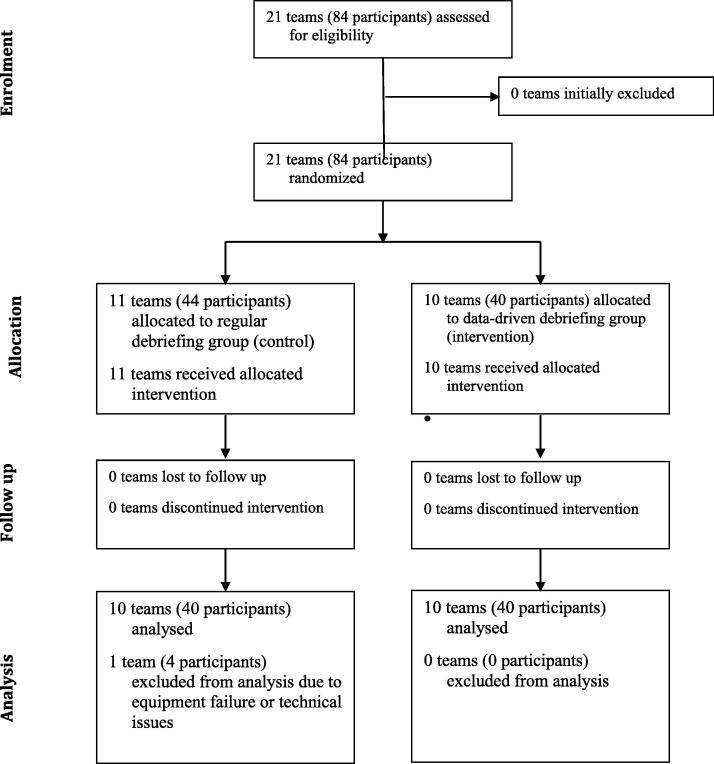
Consolidating Standards of Reporting Trials Diagram.

Twenty-one teams (84 participants) were recruited between July 2019 to December 2022. Data from one team in the control group were excluded due to technical issues (i.e. failure to save CPR quality data) (Fig. 1). Data from the remaining 20 teams (n = 10 control, n = 10 intervention) were included in the analysis. The participants had a median experience of 10 years (control 10 years; intervention 11 years). Sixty-eight (85%) were females (control 36 [90%]; intervention 32 [80%]), and 22 (27.5%) were physicians (control 11 [27.5%]; intervention 11 [27.5%]). (Table 2).

**Table 2:** Demographic characteristics.

		Control n = 40	Intervention n = 40
Gender	Female	36 (90.0)	32 (80.0)
	Male	4 (10.0)	8 (20.0)
Profession	Nurse	29 (72.5)	27 (67.5)
	Respiratory therapist	0 (0.0)	2 (5.0)
	Physician	11 (27.5)	11 (27.5)
Experience (year)	Median (IQR)	10 (7–14)	11 (5–17)
Resuscitation Course^a^	Instructor	4 (10.0)	5 (12.5)
	Taken within 1 month	4 (10.0)	1 (2.5)
	Taken within 1–6 months	17 (42.5)	17 (42.5)
	Taken within 7–12 months	9 (22.5)	12 (30.0)
	Taken more than 12 months ago	5 (12.5)	5 (12.5)
	I prefer not to answer	1 (2.5)	0 (0.0)
Chest compression on ***real*** pediatric patient	Never	14 (35.0)	15 (37.5)
	Less than 5 times	19 (47.5))	16 (40.0)
	Five times or more	6 (15.0)	9 (22.5)
	Prefer not to answer	1 (2.5)	0 (0.0)
Chest compression on ***simulated*** pediatric patient	Never	4 (10.0)	3 (7.5)
	Less than 5 times	12 (30.0)	8 (20.0)
	Five times or more	22 (55.0)	29 (72.5)
	Prefer not to answer	2 (5.0)	0 (0.0)
Feedback device used in ***real*** resuscitation event	No	18 (45.0)	23 (57.5)
	Yes	20 (50.0)	17 (42.5)
	Prefer not to answer	2 (5.0)	0 (0.0)

a. Resuscitation courses include Basic Life Support (BLS), Advanced Cardiovascular Life Support (ACLS), Pediatric Advanced Life Support (PALS), and Pediatric Emergency Assessment Recognition and Stabilization (PEARS).

IQR: interquartile range.

### CPR quality

**Fig. 2:**
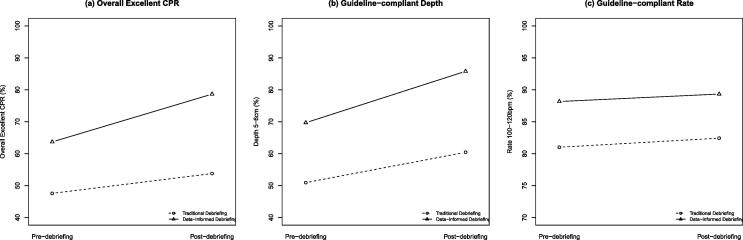
Quality of CPR in Traditional vs. Data-informed Debriefing Groups – (a) Overall excellent CPR; (b) Guideline-compliant depth; (c) Guideline-compliant rate. The data-informed debriefing group showed greater improvement in overall excellent CPR and compliance with CPR depth guidelines compared to the traditional debriefing group.

CPR quality metrics in pre-debriefing session (baseline performance) were similar between the 2 study groups (Table 3). Although both groups improved their performance after the debriefing, the data-informed debriefing group was significantly better in percentage of overall excellent CPR (control vs intervention: 53.8% vs 78.7%; Mean difference [MD] 24.9%, 95% confidence interval [CI]: 5.4 to 44.4%, *p* = 0.02) and guideline-compliant depth (control vs. intervention: 60.4% vs 85.8%, MD 25.4%, 95%CI: 5.5 to 45.3%, *p* = 0.02) compared to the control group. The difference in percentage of guideline-compliant rate was not statistically significant (Table 3). After adjusting for pre-debriefing performance, the difference in overall excellent CPR remained statistically significant (*p* = 0.04) (Table 3, Fig 2.).

**Table 3:** Clinical performance in simulated pediatric cardiac arrest.

	**Pre-debriefing (Baseline)**				**Post-debriefing**				**Post-debriefing difference adjusting for baseline performance**	
	**Control** **Mean (SD)**	**Intervention Mean (SD)**	**MD** **(95%CI)**	**P-value**	**Control** **Mean (SD)**	**Intervention Mean (SD)**	**MD** **(95%CI)**	**P-value**	**MD** **(95%CI)**	**P-value**
***CPR quality***										
Overall excellent CPR (%)	47.6 (26.9)	63.7 (11.1)	16.1 (−4.0, 36.2)	0.11	53.8 (26.5)	78.7 (9.2)	24.9 (5.4, 44.4)	0.02	11.7 (0.6, 22.8)	0.04
Depth 5–6 cm (%)	50.9 (27.5)	69.7 (8.3)	18.8 (−1.3, 38.8)	0.06	60.4 (27.6)	85.8 (4.8)	25.4 (5.5, 45.3)	0.02	9.3 (−0.9, 19.6)	0.07
Rate 100–120 bpm (%)	81.0 (10.6)	88.2 (7.5)	7.2 (−1.5, 15.8)	0.10	82.4 (17.3)	89.3 (8.1)	6.9 (−6.1, 20.0)	0.27	1.8 (−10.6, 14.2)	0.76
***Pauses in CPR***										
Chest compression fraction (%)	80.1 (9.1)	83.6 (9.9)	3.5 (−5.4, 12.4)	0.42	88.6 (4.6)	92.6 (1.9)	4.0 (0.5, 7.4)	0.03	3.4 (0.3, 6.5)	0.03
Peri-shock duration (sec)	7.6 (3.8)	7.1 (3.5)	−0.5 (−4.0, 3.0)	0.76	5.8 (1.6)	3.7 (1.4)	−2.1 (−3.5, −0.8)	0.004	−2.1 (−3.5, −0.7)	0.006
***Time to critical tasks***										
Time to initiate CPR (sec)	39.4 (21.6)	27.4 (12.1)	−12.0 (−28.4, 4.4)	0.17	16.0 (11.1)	12.1 (7.0)	−3.9 (−12.6, 4.8)	0.36	−0.6 (−8.8, 7.6)	0.88
Time to first defibrillation (sec)	160.0 (119.2)	109.3 (53.2)	−50.7 (−137.4, 36.1)	0.24	74.5 (39.3)	57.6 (28.6)	−16.9 (−49.3, 15.5)	0.39	−7.2 (−37.1, 22.7)	0.63
Time to first IV epinephrine (sec)	176.0 (41.9)	189.4 (61.9)	13.4 (−36.3, 63.1)	0.58	115.7 (58.6)	107.8 (42.7)	−7.9 (−56.0, 40.3)	0.73	−13.9 (−58.5, 30.7)	0.52

SD: standard deviation; MD: mean difference; CI: confidence interval; CPR: cardiopulmonary resuscitation.

### Chest compression fraction and pause duration

**Fig. 3:**
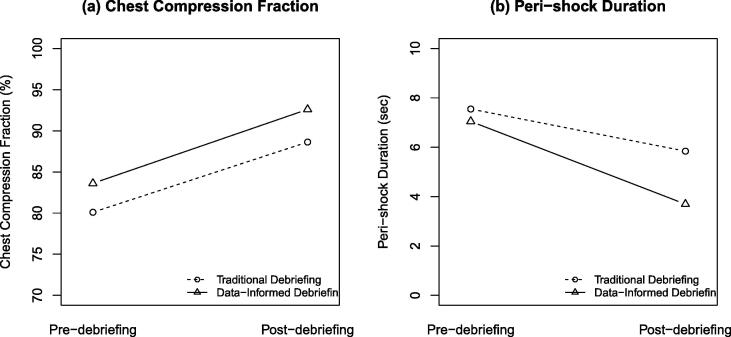
Pauses in CPR in Traditional vs. Data-informed Debriefing Groups – (a) Chest compression fracture; (b) Peri-shock duration. The data-informed debriefing group showed greater improvement in chest compression fraction and peri-shock pause duration compared to the traditional debriefing group.

The data-informed debriefing group performed significantly better with a higher CC fraction (control vs intervention: 88.6% vs 92.6, MD 4.0%, 95%CI: 0.5 to 7.4%, *p* = 0.03) compared to the control group. This difference remained significant after adjusting for pre-debriefing performance (*p* = 0.03) (Table 3). The peri-shock duration was not statistically significantly different at baseline between the two groups. Both groups improved after the debriefing. The group receiving data-informed debriefing significantly outperformed the group receiving traditional debriefing in peri-shock duration (control vs intervention: 5.8 s vs 3.7 s, MD −2.1 s, 95%CI: −3.5 to −0.8 s, *p* = 0.004), with the difference remaining significant after adjusting for pre-debriefing performance (*p* = 0.006) (Fig. 3).

### Time to critical interventions

Compared to control group, the time to critical interventions metrics in the data-informed debriefing group were decreased, but the differences were not statistically significant for time to initiate CPR (control vs intervention: 16.0 s vs 12.1 s, MD: −3.9 s, 95%CI: −12.6 to 4.8 s, *p* = 0.36), first defibrillation (control vs intervention: 74.5 s vs 57.6 s, MD: −16.9 s, 95%CI: −49.3 to 15.5 s, *p* = 0.39) and first dose of epinephrine (control vs intervention: 115.7 s vs 107.8 s, MD: −7.9 s, 95%CI: −56.0 to 40.3 s, *p* = 0.73) (Table 3).

## Discussion

To our knowledge, this is the first study comparing structured, data-informed debriefing with traditional debriefing and reporting critical performance metrics during simulated cardiac arrest care. Although traditional debriefing improves clinical performance, our study has shown additional benefit when using objective data supported by a debriefing tool during clinical event debriefing. The results of this study suggested that data-informed debriefing significantly improves several important CPR quality metrics during simulated resuscitation.

Central to the task of debriefing is the ability to accurately recognize performance gaps.[23], [40], [41] Traditional debriefing relies on learner self-assessment and facilitator observations to identify performance gaps. Previous literature suggests that code leaders often fail to accurately recall errors during resuscitation events.^21^ Even with the presence of real-time feedback, the majority of practicing healthcare providers overestimated the quality of CPR delivered during simulated pediatric cardiac arrest events.[20], [42] Unfortunately, this evidence suggests that learner self-assessment and facilitator observations are often flawed, leading to performance gaps being inadequately addressed or omitted from discussion during traditional debriefing. These patterns help to explain why CPR performance showed limited improvement in the traditional debriefing group. Data-informed debriefing directly addresses this issue by providing accurate, objective data for all key performance metrics, which helps to frame the ensuing discussion of each performance gap.

The debriefing tool used in this study includes two important features which we believe helped to improve clinical performance: (1) a list of key clinical tasks and clinical targets; and (2) a summary of teaching points for each clinical task. Use of a debriefing tool has been shown to decrease the cognitive load of facilitators and improve learner knowledge acquisition and performance.[22], [43] With the support of objective performance data, use of a debriefing tool ensures that facilitators will systematically review the performance of all key clinical tasks relative to targets and provide actionable solutions to improve the performance of each team. The teaching points provided in the debriefing tool are supported by existing evidence – for example, we teach the team leader to share their mental model prior to defibrillation as this behavior is associated with reduced peri-shock pause duration during cardiac arrest.^29^ In summary, the debriefing tool helps to ensure that all key clinical tasks are discussed, with performance gaps address in a standardized, evidence-based fashion.

Use of the debriefing tool also helped debriefers implement a ‘learning from success’ approach to the debriefing.^44^ Many debriefers are taught a ‘deficit-oriented, corrective approach’^44^ which focuses entirely on identifying and closing performance gaps. We sought to supplement this approach by reinforcing good performance, thus ensuring that all participants understand why and how good performance was achieved.^45^ To ensure this ‘learning from success’ approach was implemented in the data-informed debriefing group, the debriefers were trained to review and discuss all key tasks (and associated teaching points) regardless of how the group performed. As a consequence of this approach, we saw groups that performed well in the first scenario (e.g. CC fraction >85%) improve their performance to even higher standards (e.g. CC fraction >90%).

In our study, we found that data-informed debriefing did not significantly improve the time to critical interventions during management of simulated pediatric cardiac arrest. The differences in time to critical interventions were all less than 1 minute, a threshold which has been previously associated with improved outcomes from cardiac arrest for these important tasks.[10], [12] Our failure to demonstrate differences in these outcomes could be partly explained by our study design. Study participants were aware that both the pre- and post-debriefing scenarios were going to be pediatric cardiac arrest, thus potentially influencing their performance during these scenarios. Also, the second scenario was conducted immediately after the debriefing; any tips provided during the debriefing were fresh on their minds heading into the second scenario. This helps to explain why participants in both groups significantly improved the time to critical tasks after debriefing.

### Limitations

Our study has several limitations. First, the sample size estimation was based on the primary outcome (i.e. overall excellent CPR). Although we demonstrated significant results for CPR quality, the sample size was likely not large enough to detect differences between the groups for time to critical interventions. Future study should focus on examining the effect of data-driven debriefing on other important advanced life support interventions (e.g. time to intubation, time to treat reversible causes of cardiac arrest). Second, the post-debriefing scenario happened immediately after the debriefing, which did not allow us to examine long-term skill retention after debriefing. Due to the nature of the intervention, it was not possible to blind the debriefers to group allocation. This may have introduced bias, as debriefers may have modified their teaching points depending on the group they were debriefing. We attempted to somewhat mitigate bias by ensuring debriefers performed both the traditional and data-informed debriefings in a standardized fashion. Lastly, participants in our study were acute care providers from a single pediatric tertiary care center in Canada, who have extensive exposure to cardiac arrest simulation research and education. This could potentially influence the generalizability of the study.

## Conclusion

When compared with traditional debriefing, data-informed debriefing supported by a debriefing tool improves CPR quality and reduces pauses in CPR during simulated pediatric cardiac arrest but does not improve time to critical interventions. Future research should explore the application of debriefing tools after real cardiac arrest events.

## Funding/Support

This study was funded by a research grant provided by the Alberta Children’s Hospital Research Institute and the Alberta Children’s Hospital Foundation.

## Role of the Funder/Sponsor

The funding agencies had no role in the design and conduct of the study; collection, management, analysis and interpretation of data; preparation, review or approval of the manuscript; and decision to submit the manuscript.

## Declaration of Competing Interest

The authors declare the following financial interests/personal relationships which may be considered as potential competing interests: Dr. Cheng reports grants from Alberta Children’s Hospital Research Institute and the Alberta Children’s Hospital Foundation during the conduct of the study. Dr. Adam Cheng is a volunteer for the American Heart Association (Resuscitation Education Writing Group) and the International Liaison Committee for Resuscitation (Vice Chair; EIT Task Force). Dr. Cheng is faculty with The Debriefing Academy, which provides debriefing courses for healthcare educators. Dr. Yiqun Lin is a volunteer for the American Heart Association (Resuscitation Education Writing Group) and the International Liaison Committee for Resuscitation (Member; EIT Task Force). The authors have no other relevant financial disclosures or conflicts of interest to declare.
